# Prevalence and causes of hearing impairment: a cross-sectional study in Karnali Province, Nepal

**DOI:** 10.1017/S0022215125000192

**Published:** 2025-08

**Authors:** Rishi Bhatta, Sailesh Kumar Mishra, Diego Santana-Hernández, Anil Paudel, Sanju Maharjan, Roshana Kandel, Ranjan Shah, Krishna Khadka, Manisha Budhathoki, Bijaya Guragain, Man Bahadur Kunwar, Manish Gautam

**Affiliations:** 1 Department of ENT, KIST Medical College and Teaching Hospital, Lalitpur, Nepal; 2 Anweshan Pvt. Ltd, Lalitpur, Nepal; 3 Nepal Netra Jyoti Sangh, Kathmandu, Nepal; 4 Christoffel-Blindenmission Christian Blind Mission e.V., Inclusive Health Initiative·IHI, Germany; 5 CBM Global, Lalitpur, Nepal

**Keywords:** hearing impairment, prevalence, severity of illness, ear diseases, etiology

## Abstract

**Objective:**

This research aims to assess the prevalence, severity and underlying causes of hearing impairments.

**Methods:**

This cross-sectional study used multistage stratified sampling to select 2148 individuals from Salyan and Surkhet, following the World Health Organization’s Ear and Hearing Survey Handbook.

**Results:**

Among 1946 participants, 38.9 per cent had hearing impairments, including 15.9 per cent with disabling hearing loss, with severity increasing with age. Ear diseases affected 34.3 per cent, including dull or retracted tympanic membranes (18 per cent), impacted wax (8 per cent), perforated tympanic membrane (6.1 per cent), and abnormal tympanometry (23.1 per cent). The major causes were age-related hearing loss (50.5 per cent), Eustachian tube dysfunction (23 per cent), chronic suppurative otitis media (10.8 per cent), and otitis media with effusion (4.7 per cent). Higher education and immunisation were associated with reduced risk, while chronic conditions, earaches, drainage and tinnitus increased the risk.

**Conclusion:**

The high prevalence of hearing impairment, primarily from preventable causes, underscores the importance of early screening and strengthened primary health care.

## Introduction

Hearing impairment is a global public health concern. Estimates indicate that more than 5.5 per cent of the population worldwide has moderate or worse hearing loss,^1^ with about 90 per cent living in low- and middle-income countries, where resources to help are often limited.^2^ Although hearing impairment often shows no visible signs, it affects around 6.1 per cent of the global population with disabling hearing loss.^3^ The World Health Organization (WHO) projects that by 2050, nearly 2.5 billion people will experience hearing loss of mild or higher severity in the better hearing ear.^4^

In Southeast Asia, 400 million people live with varying degrees of hearing loss, representing 25 per cent of the global cases.^5^ According to Nepal’s 2021 census, 15.6 per cent of the country’s population has a hearing disability, representing 2.2 per cent of total disabilities. The National Statistics Office (NSO) also found that 6.3 per cent of people in Nepal have speech impairment.^6^ The Nepal Burden of Disease 2017 report highlights that nearly 4 per cent of ‘Years Lived with Disability’ comes from age-related hearing loss, which has risen by 31 per cent since 1990.^7^

Hearing impairment is both a cause and consequence of poverty, especially in low- and middle-income countries.^8^ Ear health and hearing are shaped by a range of genetic, biological, psychological, and environmental factors throughout life, from prenatal to old age. Additionally, non-modifiable risk factors such as age, gender, congenital factors, medical conditions, loud noises, medications, diet, and occupational hazards all play roles in ear health. The occurrence, severity, and progression of hearing loss depend on the interaction of these factors.^9^ When not identified early or effectively treated, hearing impairment can hinder communication, language development, education, social life, cognitive function, and overall quality of life, leading to substantial costs for both individuals and society.^1^ In Nepal, hearing loss appears to be more common than in other developing countries, possibly due to factors such as lower socio-economic status, limited awareness, and inadequate healthcare facilities, particularly in rural areas.^10^

Nepal currently lacks a national survey on ear and hearing health. However, regional studies consistently show a high prevalence of hearing loss, exceeding WHO’s threshold of 4 per cent, highlighting it as an urgent public health issue.^11^ In Karnali Province, one of Nepal’s most marginalised regions with a hard-to-reach population, the status of individuals with hearing difficulties remains unidentified. This study, therefore, aimed to assess the prevalence of hearing impairment in Surkhet and Salyan districts of Karnali Province and to identify potential causes.

## Materials and methods

The cross-sectional study was conducted in six municipalities (three in each of Salyan and Surkhet districts) from 17 February to 16 March 2024. The research followed the guidelines outlined in WHO’s Ear and Hearing Survey Handbook (2020), which was designed to estimate hearing impairment prevalence, including age- and gender-specific rates, to assess different grades of hearing loss according to WHO standards, and to identify common causes of hearing loss in these districts.

### Study population

The study included individuals of all ages, backgrounds, and disabilities from randomly selected households, excluding those who had lived in the area for less than six months. To avoid selection bias, the chosen households were given a ‘yellow card’ with family details, which was required for participation. Only individuals with this card could participate, while self-reported individuals without a ‘yellow card’ were excluded.

### Sampling strategy

The study used a stratified multistage cluster sampling approach. The population was first divided into two main groups (strata) by district: Surkhet and Salyan. Within each district, local levels served as secondary strata. Each ward within a local level was treated as a separate cluster. In the first stage, the clusters were selected using the probability-proportional-to-size method. After choosing wards, each selected ward was further divided into blocks, and lists of neighbourhoods (‘toles’) and households were obtained from ward offices and organised into distinct blocks. In the second stage, one block was randomly selected from each ward using simple random sampling. A household-listing process was conducted following the block selection, and, in the third stage, a systematic random sampling technique was applied to select households within each chosen block. Finally, in the fourth stage, all eligible individuals within the selected households were included in the study.

### Sample size

Based on WHO’s 5.5 per cent prevalence of hearing loss,^1^ with a precision of 1.5 per cent, an 85 per cent participation rate, and a design effect of two to adjust for within-cluster homogeneity, the required sample size was calculated to be 2090. To ensure maximum population coverage, a 20 per cent dropout rate was added, bringing the final sample size to 2508 individuals. With a total of 78,231 households and an average family size of four, the number of households to be surveyed was 627. Given a cluster size of 30, this required selecting 21 households per cluster (i.e., 627/30).

### Sampling frame

Based on data from the 2021 National Population and Housing Census, the total population in the study area included 321,565 individuals: 93,543 in Salyan and 228,022 in Surkhet. The sample distribution across the selected municipalities, determined using the probability-proportional-to-sampling technique, is shown in Table 1.

**Table 1.** Sampling distribution for study site

Strata-Salyan			
**Local level strata**	*Total households	**Selected wards**	**Estimated sample size per cluster**
Kumakh Gaunpalika	5491	1,4	42
Bagachour Municipality	7498	1,4,8	63
Sharada Municipality	8898	1,4,9,14	84
**Total**	**21,887**	**9**	**189**
**Strata-Surkhet**			
Gurbhakot Municipality	11,798	3,6,9,12	84
Birendranagar Municipality	38,377	1,2,3,4,5,6,7,9,10,11,12,14	315
Barahatal Gaunpalika	6169	4,7	42
**Total**	**56,344**	**21**	**441**

* National Population and Housing Census 2021; wards 3, 9, and 12 selected twice under the probability-proportional-to-size method.

### Study tool and instruments

The survey tool was developed following the WHO’s Ear and Hearing Survey Handbook guidelines and included sections on demographic information and exposure to risk factors. The study also included clinical assessments, such as otoscopic examinations, tympanometry, and audiological tests such as otoacoustic emissions (OAE) and pure-tone audiometry (PTA). These tests were used to identify and measure the extent of hearing loss and ear diseases.

### Research team and training

An eight-member team consisting of one ENT specialist, two ear and hearing care workers, three researchers, and two local volunteers conducted the fieldwork over 31 days. Prior to data collection, the ear and hearing care workers and researchers underwent an eight-day online training program and a two-day practical training session. This training was based on WHO’s standard protocol for intermediate-level proficiency and covered topics from modules 1–9 of the ‘Primary Ear and Hearing Care Training’ manual, with some adjustments for local context.^13^

### Pilot study and presurvey visit

A pilot study was carried out in Bheriganga Municipality, Surkhet, in similar settings. Feedback from the field team and participants was collected and used to adjust and improve the survey tool before starting the main survey. A pre-survey visit was also conducted to prepare for the actual survey, during which the study team coordinated with municipal and ward offices, gathered information on neighbourhoods and households, formed blocks, randomly selected blocks within clusters, conducted social mapping, and finalised the campsite location.

### Data collection

Before data collection, local volunteers created a household roster using the KoboToolbox (Cambridge, MA) software to ensure all eligible individuals were included. They explained the study’s objectives to households and encouraged them to attend a designated camp. Data collection involved community-based camps, household visits, or a mixed approach to minimise dropout rates. Data collection was conducted in three phases: face-to-face interviews for socio-demographic and medical history, hearing assessments and ear examinations.

#### Hearing assessment

Before beginning the hearing assessment, ambient noise levels were checked and kept below 40 dBA. Children aged 0–4 years underwent OAE testing, while those aged five and above took PTA tests using the Arphi Proton Dx3 audiometer (Arphi Electronics, Mumbai, India). Air conduction thresholds for both ears were measured at 500Hz, 1000Hz, 2000Hz and 4000Hz and recorded in the personalised data collection form.

#### Ear examination and tympanometry

After the hearing tests, patients were moved to a designated room for a comprehensive ear examination. An otoscope was used to inspect the ear canal and tympanic membrane for any abnormalities or signs of disease. Tympanometry was conducted to assess middle-ear function and detect issues related to the eardrum, middle-ear cavity and ossicles.

**Figure 1.**
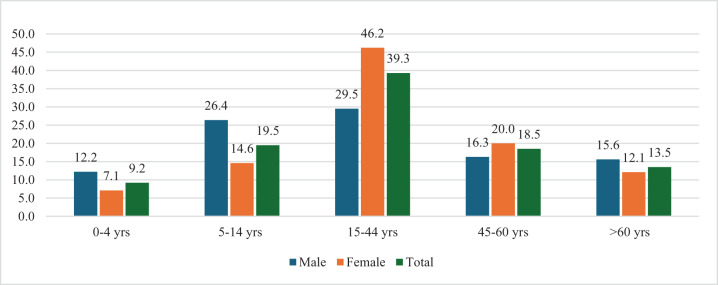
Distribution of participants by age and gender.

### Data management and analysis

After fieldwork was completed, trained individuals, following a proper orientation, entered the data into the system using KoboToolbox. Supervisors then thoroughly checked, reviewed and corrected the uploaded data. Next, data cleaning and validation were conducted to rectify errors or inconsistencies. The data were then transferred to SPSS software (IBM, Armonk, NY) for management and analysis, and syntax was set up for the survey dataset.

In the analysis, both descriptive and inferential statistics were used to understand the prevalence, causes, and risk factors for hearing loss among people in the study area. Frequencies and percentages were calculated for the descriptive part. Inferential statistics included the chi-square test and logistic regression. Only variables that showed significant associations in univariate analysis (these included variables with a *p*-value below 0.05 at a 95 per cent confidence interval) were included in the multivariate analysis.

**Table 2.** General ear and hearing health status

Characteristics	Frequency	Percentage
**Tinnitus experience**		
– Yes	781	36.4%
– No	1112	51.8%
– Uncertain	255	11.9%
**History of earache**		
– Yes	591	27.5%
– No	1496	69.6%
– Uncertain*	61	2.8%
**History of discharging/draining ears**		
– Yes	257	12.0%
– No	1891	88.0%
**History of ear surgery**		
– Yes	34	1.6%
– No	2114	98.4%
**Immunisation coverage**		
– Yes	1197	55.7%
– No	431	20.1%
– Uncertain	520	24.2%
**Have relatives with difficulty in hearing**		
– Yes	320	14.9%
– No	1821	84.8%
– Uncertain	7	0.3%
**Experience of ototoxic effect on hearing**		
– Yes	33	1.5%
– No	1665	77.5%
– Uncertain	111	5.2%
– Not applicable	339	15.8%
**Exposure to loud sound**		
– Never	225	10.5%
– Once a month or less	375	17.5%
– 2−3 times per month	404	18.8%
– Once a week	383	17.8%
– Almost every day	761	35.4%
**Use of headphones**		
– Never	1594	74.2%
– Once a week or less	102	4.7%
– 2−3 times per week	157	7.3%
– Everyday	56	2.6%
– As per need	41	1.9%
– Not applicable	198	9.2%

* used in cases of children who could not report by themselves.

### Definition of hearing impairment

In accordance with the WHO’s Ear and Hearing Survey Handbook, hearing impairment was calculated using a threshold of above 20 dB HL in the better ear. Grades of hearing loss were determined based on the average hearing level in the better ear at 0.5, 1, 2, and 4 kHz on PTA, as follows: mild >20 to <35 dB HL; moderate 35 to <50 dB HL; moderate to severe 50 to <65 dB HL; severe 65 to <80 dB HL; profound 80 to <95 dB HL; complete ≥95 dB HL. Unilateral hearing loss was identified when the better ear measured below 20 dB HL and the worse ear measured 35 dB HL or more.^12^

### Ethical consideration

The Nepal Health Research Council (NHRC) approved the study, with protocol registration number 689/2023, on 9 January 2024. Before data collection began, informed consent was obtained from all participants, who were also told they could leave the study at any time without any consequences. Confidentiality and anonymity were rigorously upheld throughout the study. In addition, since the survey included individuals of all ages from selected households, parental consent was obtained for participants under 18 years of age.

## Results

### Socio-demographic information

Out of the 2148 study participants, 58.8 per cent were female (see Figure 1). The predominant age group was 15–44 years, making up 39.3 per cent of the total. Notably, females outnumbered males in this group by 17 per cent.

### General ear and hearing health status

More than one-third (36.4 per cent) of participants experienced tinnitus, which affected their sleep (59.7 per cent), concentration (56.0 per cent), work (56.6 per cent) and daily activities (39.6 per cent) (see Table 2). The study also found that 27.5 per cent had a history of frequent earaches, 12 per cent experienced ear discharge and 1.6 per cent had already undergone ear surgery. Over half (55.7 per cent) were immunised according to the national schedule, and 14.9 per cent had relatives with hearing difficulties. Only 1.5 per cent reported hearing loss possibly due to ototoxic drugs. In addition, 35.4 per cent had daily exposure to loud sounds, and 16.5 per cent used headphones at varying levels.

### Prevalence and distribution of hearing impairment in different age groups

A total of 198 children under five years of age underwent OAE testing to assess their hearing capacity (see Table 3). Of these, 80.3 per cent passed the test, while the remaining 19.7 per cent were referred for further evaluation.

**Table 3.** Otoacoustic emissions (OAE) test for children 0–4 years of age

OAE test	Male		Female		Total	
	*n*	%	*n*	%	*n*	%
Pass	84	77.8%	75	83.3%	159	80.3%
Refer	24	22.2%	15	16.7%	39	19.7%
**Total**	**108**	**100.0%**	**90**	**100.0%**	**198**	**100.0%**

Four participants over five years of age were unable to undergo PTA due to intellectual disability. Of the remaining 1946 participants who underwent PTA, 38.9 per cent had hearing impairment (see Table 4). Mild hearing impairment affected 19.6 per cent of participants, with only four experiencing complete hearing loss or deafness. Age was significantly associated with hearing loss (χ^2^ = 855.471, *p*-value: 0.000). Age analysis showed that 93.8 per cent of individuals over 60 years of age exhibited some degree of hearing loss, followed by 62 per cent in the 45–60 years old age group, 23.5 per cent in the 15–44 years old age group, and 10.1 per cent in the 5–14 years old age group.

**Table 4.** Prevalence and distribution of hearing impairment in different age groups (n = 1946); chi-square (χ^2^) = 855.471, p-value = 0.000

Grades of hearing impairment	5−14 years	15−44 years	45−60 years	>60 years	Total %	*n*
Mild	5.3%	14.6%	34.5%	34.0%	19.6%	381
Moderate	2.6%	3.3%	15.6%	32.3%	10.0%	195
Moderate to severe	–	1.4%	6.8%	14.8%	4.2%	82
Severe	–	0.7%	1.8%	9.6%	2.1%	41
Profound	–	–	0.8%	2.1%	0.5%	9
Complete	–	0.2%	0.3%	0.3%	0.2%	4
Unilateral	2.2%	3.2%	2.3%	0.3%	2.4%	46
Overall prevalence	10.1%	23.5%	62.0%	93.8%	38.9%	758

### Distribution of hearing impairment according to gender differences

Although the overall prevalence of hearing impairment shows no significant gender differences in the bivariate analysis, specific age groups showed variations. Females generally have a higher prevalence of mild hearing impairment than males, except in the 5–14 years old age group, with the total proportion in females exceeding males by 12.2 per cent. In contrast, males have a higher prevalence of moderate hearing impairment across all age groups except 5–14 years old, with females having 8.6 per cent less moderate impairment overall (see Table 5).

**Table 5.** Distribution of hearing impairment (HI) according to gender differences in different age groups (n = 758)

Grades of HI	5−14 years		15−44 years		45−60 years		>60 years		Total	
	Male	Female	Male	Female	Male	Female	Male	Female	Male	Female
Mild	60.0%	41.2%	48.1%	67.4%	52.7%	57.4%	30.5%	41.8%	42.9%	55.1%
Moderate	24.0%	29.4%	24.1%	10.4%	25.3%	25.2%	38.9%	30.5%	30.9%	22.3%
Moderate to severe	–	–	11.1%	4.2%	16.5%	7.7%	17.6%	14.2%	14.6%	8.3%
Severe	–	–	1.9%	3.5%	4.4%	1.9%	9.2%	11.3%	5.6%	5.3%
Profound	–	–	–	–	–	1.9%	3.1%	1.4%	1.3%	1.1%
Complete	–	–	1.9%	0.7%	1.1%	–	–	0.7%	0.7%	0.4%
Unilateral	16.0%	29.4%	13.0%	13.9%	–	5.8%	0.8%	–	4.0%	7.4%
**Total**	**25**	**17**	**54**	**144**	**91**	**155**	**131**	**141**	**301**	**457**

### Disabling hearing loss

Participants with hearing loss greater than 35 dB HL in the better ear were classified as having disabling hearing loss, with a prevalence of 15.9 per cent. The prevalence rate increased with age, reaching 57 per cent in those above 60 years of age. When disaggregated by gender, males were 5.5 per cent more affected by disabling hearing loss than females. In all age groups except 5–14 years old, males had a higher prevalence of disabling hearing loss (see Table 6).

**Table 6.** Prevalence of disabling hearing loss according to age and sex differences

Age group	Gender	Disabling	Non-Disabling
5−14 years old	Male	1.3%	98.7%
	Female	2.7%	97.3%
	Subtotal	1.9%	98.1%
15−44 years old	Male	7.7%	92.3%
	Female	4.0%	96.0%
	Subtotal	5.1%	94.9%
45−60 years old	Male	27.1%	72.9%
	Female	20.9%	79.1%
	Subtotal	23.2%	76.8%
>60 years old	Male	63.0%	7.6%
	Female	51.6%	48.4%
	Subtotal	57.0%	43.0%
Overall	Male	19.2%	80.8%
	Female	13.7%	86.3%
	**Total**	**15.9%**	**84.1%**

### Ear diseases

An otoscope and tympanometry were used to examine participants’ ears, revealing that 737 individuals (34.3 per cent) had ear diseases in either one or both ears. Among the findings, 0.7 per cent had malformed auricles, such as pre-auricular sinus, 0.5 per cent had ear trauma, and 0.1 per cent had a pinna infection. Wax was present in 19.6 per cent, with 8 per cent having impacted wax. Foreign bodies were found in 0.7 per cent, and 0.2 per cent had inflammation of the external ear canal. Otorrhoea (the discharge of fluid or pus) and fungal infections affected 1.9 per cent and 1.8 per cent, respectively, in the external ear canal. A dull or retracted tympanic membrane was observed in 18 per cent, and 6.1 per cent had a perforated tympanic membrane. A red and bulging tympanic membrane, which indicates upper respiratory infection, chronic allergies, or sinusitis, was seen in 2.3 per cent. Otorrhoea in the middle ear affected 2.2 per cent, and cholesteatoma was found in 0.6 per cent. Abnormal tympanometry results were observed in 23.1 per cent of participants (see Table 7).

**Table 7.** Ear diseases found in either or both ears (*n* = 2148)

Conditions	Frequency	Percentage
**Pinna**		
– Auricle malformation	14	0.7%
– Auricle trauma	11	0.5%
– Auricle infection	2	0.1%
**External Ear Canal**		
– Impacted wax	171	8.0%
– Foreign body (FB)	15	0.7%
– Inflammation of ear canal	5	0.2%
– Otorrhea of external ear	40	1.9%
– Fungus (Otomycosis)	38	1.8%
**Tympanic membrane**		
– Perforation	131	6.1%
– Dull and retracted	386	18.0%
– Red and bulging	50	2.3%
**Middle ear**		
– Otorrhea	48	2.2%
– Cholesteatoma	13	0.6%
– Abnormal tympanometry	497	23.1%

### Probable cause of hearing loss

The probable causes of hearing loss were analysed based exclusively on the threshold of the better ear. The primary cause of hearing loss at any level was age-related hearing loss, making up 50.5 per cent, followed by Eustachian tube dysfunction at 23 per cent, chronic suppurative otitis media (CSOM) at 10.8 per cent, wax impaction at 7 per cent, otitis media with effusion (OME) at 4.7 per cent, and sensorineural hearing loss (SNHL) at 2.2 per cent (see Table 8). However, the cause of hearing loss was unknown for 5.4 per cent of participants. It was observed that Eustachian tube dysfunction was mainly associated with mild to moderate hearing loss, while age-related hearing loss was linked to moderate, moderate to severe, severe, and profound hearing loss. Similarly, idiopathic SNHL was most often associated with complete hearing loss.

**Table 8.** Probable causes and grades of hearing loss amongst those with any level of hearing loss (*n* = 758)

Causes	Mild	Moderate	Moderate to severe	Severe	Profound	Complete	*Unilateral	Total frequency	Total percentage
Unknown	8.1%	1.5%	–	2.4%	–	25.0%	10.9%	41	5.4%
Wax	6.8%	9.2%	7.3%	2.4%	11.1%	–	2.2%	53	7.0%
CSOM	7.6%	8.2%	13.4%	17.1%	33.3%	–	34.8%	82	10.8%
OME	5.2%	5.1%	2.4%	2.4%	–	–	6.5%	36	4.7%
AOE	0.3%	0.5%	–	–	–	–	–	2	0.3%
ASOM	1.0%	0.5%	–	–	–	–	–	5	0.7%
FB	–	0.5%	–	–	–	–	–	1	0.1%
ARHL	36.5%	70.8%	78.0%	75.6%	88.9%	25.0%	4.3%	383	50.5%
SNHL	0.8%	2.6%	3.7%	2.4%	–	25.0%	8.7%	17	2.2%
Congenital	–	–	–	4.9%	–	50.0%	–	4	0.5%
NIHL	0.5%	0.5%	1.2%	–	–	–	–	4	0.5%
ETD	35.2%	11.3%	3.7%	2.4%	–	–	30.4%	174	23.0%
Otosclerosis	–	1.0%	–	–	–	–	–	2	0.3%
Sequelae of Otitis media	0.5%	1.0%	–	–	–	–	2.2%	5	0.7%
Cerebral palsy	–	–	1.2%	–	–	–	–	1	0.1%
***n***	**381**	**195**	**82**	**41**	**9**	**4**	**46**	**758**	**100.0**

* cause in better ear; CSOM = chronic suppurative otitis media; OME = otitis media with effusion; AOE = acute otitis externa; ASOM = acute suppurative otitis media; FB = foreign body; ARHL = age-related hearing loss; SNHL = sensorineural hearing loss; NIHL = noise-induced hearing loss; ETD = Eustachian tube dysfunction.

### Risk factors analysis

The variables (age, gender, education status, income, smoking, chronic medical conditions, immunisation status, family history of hearing loss, ototoxic effects, use of headphones, exposure to loud sound, history of earache, history of ear drainage and tinnitus) were initially included in the model. However, only those with a *p*-value of 0.05 or less were retained. Age, considered a confounding factor, was excluded from the final analysis. Thus, the final model included education status, chronic medical conditions, immunisation status, history of earache, smoking habits, history of ear draining, and tinnitus experience.

Higher education reduced the likelihood of hearing impairment by 91.2 per cent compared to illiteracy (odds ratio: 0.088; 95 per cent confidence interval (CI): 0.052–0.150) (see Table 9). Similarly, secondary (odds ratio: 0.147; 95 per cent CI: 0.091–0.238) and primary education (odds ratio: 0.159; 95 per cent CI: 0.103–0.245) were associated with a lower likelihood of hearing impairment. Participants with chronic medical conditions had 3.264 times higher odds of hearing impairment (odds ratio: 3.264; 95 per cent CI: 2.165–4.922). Immunised participants following the national schedule were 51 per cent less likely to experience hearing impairment (odds ratio: 0.490; 95 per cent CI: 0.368–0.650). Those with a history of frequent earaches had 1.512 times higher odds of hearing impairment (odds ratio: 1.512; 95 per cent CI: 1.120–2.042). Participants with draining ears had 2.360 times higher odds of hearing impairment (odds ratio: 2.360; 95 per cent CI: 1.604–3.471), and those with tinnitus had 2.019 times higher odds (odds ratio: 2.019; 95 per cent CI: 1.543–2.642). Smoking did not show a significant association in multivariate analysis.

**Table 9.** Multivariate analysis of risk factors; CI = confidence interval

Factors	Hearing impairment		
	AOR	95% CI	*p*-value
**Education**			
– Higher secondary	0.088	0.052−0.150	0.000
– Secondary	0.147	0.091−0.238	0.000
– Primary	0.159	0.103−0.245	0.000
– Illiterate	Ref		
*Chronic medical conditions			
– Present	3.264	2.165−4.922	0.000
– Not present	Ref		
**Immunisation status**			
– Yes	0.490	0.368−0.650	0.000
– No	Ref		
**History of earache**			
– Yes	1.512	1.120−2.042	0.007
– No	Ref		
**Habit of smoking**			
– Yes	1.452	0.910−2.314	0.117
– No	Ref		
**History of Ear draining**			
– Yes	2.360	1.604−3.471	0.000
– No	Ref		
**Tinnitus experience**			
– Yes	2.019	1.543−2.642	0.000
– No	Ref		

* Chronic medical conditions encompass diabetes, hypertension, kidney diseases, hypercholesterolemia, and rheumatic diseases. AOR = Adjusted Odds Ratio; Ref = Reference

### Action needed

Among the participants examined, 682 (31.8 per cent) required actions or services, 77 per cent of whom needed medications, 14.7 per cent required hearing aids, and 16.6 per cent were referred for further evaluation, possibly including additional diagnostics or treatment such as surgery. Only a small percentage (0.6 per cent) were urgent cases needing advanced diagnostics for medication (see Table 10).

**Table 10.** Population in need of ear and hearing services

Action needed*		Frequency	Percentage’
**Yes (682)**	Medication	525	77.0%
	Hearing aids	100	14.7%
	Referral for further evaluation	113	16.6%
	Urgent referral	4	0.6%

* Multiple response ’Might exceed 100%

## Discussion

This cross-sectional, community-based study provides insights into the prevalence and levels of hearing loss, along with etiological causes and risk factors, across all age groups in the Salyan and Surkhet districts of Karnali Province, Nepal. The study followed the WHO’s Ear and Hearing Survey Handbook, and used a mixed-data collection method through household visits and camp-based assessments. During visits and camps, participants were directed to a hearing-assessment room with ambient noise below 40 dBA after initial history-taking. Hearing assessments were carried out before any ear examination, as per protocol, because actions such as removing wax, debris, discharge, or foreign objects from the ear could have affected true hearing results, avoiding any potential bias from the audiologists.

Audiometry measurements followed WHO guidelines, averaging four air-conduction frequencies. While mobile devices can estimate hearing threshold accurately,^14^ this survey applied classical PTA tests (with battery backup). Bone conduction was not tested; hence some cases were labelled as unknown because they needed a comprehensive evaluation to determine cause.

This study also highlights tinnitus as a significant public health issue, affecting over one-third of the population. More than half of those with tinnitus reported disturbances in sleep, concentration, and work, which is a higher rate than a recent study in Nepal that reported 11.7 per cent tinnitus incidence in adults.^15^ Further research using the Tinnitus Handicap Inventory is recommended.

The study revealed a concerning prevalence of hearing impairment (38.9 per cent), which is higher than the 16.6 per cent found in a 2007 study in Nepal.^16^ However, this difference may be due to methodological disparities. The earlier study defined hearing impairment as a threshold worse than 30 dB in either ear. Additionally, the timing of the studies also may have influenced the variation in results. This survey introduced a new grading system for hearing loss in Nepal, which makes direct comparisons with past studies less relevant.

The prevalence of disabling hearing loss in this study was 15.9 per cent, which is higher than the 6.1 per cent found in the ‘WHO Ear and Hearing Disorders Survey’ in Guizhou Province, China. However, methodological differences also contributed to this variation: this study defined disabling hearing loss as greater than 35 dB in the better ear, while the Guizhou study^17^ used thresholds of more than 40 dB for adults and greater than 35 dB for children.

Our analysis showed a strong link between age and severity of hearing impairment (χ^2^: 855.471, *p*-value: 0.000), with hearing impairment increasing with age. The prevalence ranged from 23.5 per cent in the 15–44 years old age group to 93.8 per cent to those more than 60 years old. This trend aligns with results from the Guizhou Province study, which also showed a significant increase in hearing loss with age (χ^2^: 2049.866, *p* <0.01), reaching 72.6 per cent among those more than 60 years old.^17^ Similarly, a study in France showed that hearing loss prevalence increased from 3.4 per cent among individuals 18–25 years old to 73.3 per cent among those 71–75 years old.^18^

Among children 5–14 years old, hearing impairment was found in 10.1 per cent, aligning with a previous study in Nepal, which reported a 10.75 per cent prevalence in children 5–19 years old.^19^ However, this rate is higher than the 5.73 per cent prevalence found in school-aged children across 509 government schools in Nepal’s hill, mountain and Terai regions.^20^ This suggests a need for targeted preventive interventions for children.

There was no significant difference in overall prevalence of hearing loss amongst males and females. However, differences in the prevalence of mild, moderate, and moderate-to-severe hearing impairment were observed within certain age groups as males were more affected by moderate and moderate to severe levels of hearing loss for all age group except 5–14 years of age. A similar study in Jiangxi Province, China, found that females had 0.73 times lower odds of hearing loss than males.^21^ Likewise, another study in China reported that males had 2.27 times the risk of hearing loss compared to females.^17^ These contradictory findings suggest further research is needed to determine whether gender is a risk factor for hearing loss.

The prevalence of ear diseases was determined to be 34.3 per cent. This included any ear disease detected in one or both ears, with some individuals showing multiple conditions simultaneously. This prevalence rate is notably higher than the 18.8 per cent reported in a 2021 study by the Curative Services Division of the Ministry of Health and Population.^22^ The prevalence calculation was based on the presence of diseases affecting the pinna, external ear canal, tympanic membrane and middle ear. Impacted wax was identified in 8 per cent of participants, a figure higher than the 3.4 per cent prevalence documented in a 2010 study conducted in Sarlahi, Nepal.^23^ Further examination by age group showed that impacted wax was present in 14.6 per cent of participants 5–14 years old, while 6.2 per cent of participants more than 60 years old had a perforated tympanic membrane. These findings are consistent with a study from Ecuador, where 13.7 per cent of participants aged 4–15 had impacted wax, and 2 per cent of those aged 65 and above had a perforated tympanic membrane.^24^

Almost 18 per cent of study participants exhibited a dull and retracted tympanic membrane, a sign often associated with untreated middle-ear infections, nasal infections, or poor Eustachian tube function. Although this figure is slightly lower than that reported in a study from eastern Nepal,^23^ it still highlights a concerning proportion that requires attention. Additionally, 7 per cent of participants had hearing loss due to impacted wax, an easily treatable condition. With proper ear and hearing care, these conditions could have been prevented. Cholesteatoma, a potentially life-threatening condition that signifies an unsafe disease in the middle-ear cavity, was found in 13 participants. Some patients showed signs of SNHL, which requires further investigation to determine the likely cause. For about 5 per cent of participants, the cause of hearing loss was unclear and needed further audiological investigations, including tests such as bone conduction and acoustic reflex to determine if the hearing loss is sensory, conductive, or mixed. Additionally, blood and imaging tests may be necessary for accurate diagnosis.

This study identified age-related hearing loss as the most common cause of hearing loss, predominantly linked with moderate to severe hearing impairment. The ‘WHO Ear and Hearing Disorder Survey’ from Guizhou Province reported that presbycusis (age-related hearing loss) accounted for over 30 per cent of hearing loss,^17^ a slightly lower percentage than observed in this study. Although age-related hearing loss is primarily age-related, various factors can worsen its effect, including comorbidities (such as hypertension, diabetes, chronic kidney disease, and lung disease), exposure to noise, use of ototoxic drugs, stress and anxiety.^25^ Following age-related hearing loss, Eustachian tube dysfunction was the next most common cause of hearing loss identified in this study. Eustachian tube dysfunction, however, mainly resulted in mild or one-sided hearing loss. As a preventable condition, Eustachian tube dysfunction’s effects can be mitigated through proper ear and hearing care.

CSOM was the third leading cause of hearing loss in this study. Previous research in Nepal^22^ has consistently highlighted CSOM as a major contributor to hearing impairment. This contrasts with findings from developed countries, where chronic middle-ear disease (both active and inactive) is the most prevalent ear condition, with a prevalence of 5.3 per cent.^26^

Upon analysing the link between risk factors and hearing loss, this study found no significant association between gender and hearing impairment, which contrasts with a previous study that suggested males were at a higher risk than females.^24^ Education level, however, was found to have a significant association with hearing impairment, although this result contradicts another study that did not find literacy levels to be influential.^23^ Immunisation appeared to be a protective factor against hearing impairment, consistent with findings from another study in Nepal.^22^ Chronic medical conditions were also significantly associated with hearing impairment, aligning with previous research. Other studies similarly support the connection between chronic conditions, ototoxic medication, and hearing impairment.^24^^,^^27^ In this study, 14.8 per cent of participants needed hearing devices, and 16.6 per cent required further diagnostic evaluation. These findings underscore the importance of timely and appropriate diagnosis and effective treatment, as well as access to high-quality hearing devices.

- Nepal’s Karnali Province faces significant gaps in diagnostics and treatment access, compounded by limited resources and specialised workforce
- Among 1946 participants (aged ≥5 years), 38.9 per cent exhibited hearing impairment, escalating with age
- Disabling hearing loss affected 15.9 per cent of participants, with males more affected than females
- Ear pathologies were found in 34.3 per cent of individuals, with common conditions including retracted tympanic membranes, impacted wax, and tympanic membrane perforations
- Immunisation and education correlated with reduced risk, while earache, drainage, and tinnitus were linked to higher rates of hearing loss
- This study underscores urgent needs for accessible diagnostics, targeted interventions and integration of ear care into primary health systems in Nepal to address preventable and treatable hearing loss

### Strength and limitations

This study has several strengths. It is a pioneering effort, as no similar study know to us has been conducted on such a large population using robust multi-stage sampling techniques for participant selection. Validated clinical tools were employed, and the insights gained from this research can guide and enhance future studies in other regions of Nepal. The findings will aid in developing more effective interventions for ear and hearing health.

However, there were some limitations. Due to infrastructure challenges, a soundproof environment could not be established at all study sites, affecting the accuracy of hearing-threshold assessments. Additionally, conducting OAE tests on children was challenging when they were not asleep. Assessing hearing status after the removal of earwax or foreign bodies could also improve the study’s accuracy.

## Conclusion

Hearing impairment in Karnali Province represents a significant public health issue, with particularly high prevalence among older adults. These findings highlight an urgent need for targeted public-health initiatives to address hearing impairment in the province. Recommended strategies include increasing awareness about ear and hearing health, enhancing access to audiological services, and implementing preventive measures such as immunisation and noise control. Additionally, the high prevalence of treatable conditions such as CSOM, Eustachian tube dysfunction, and wax impaction also indicates that strengthening primary healthcare services could significantly reduce the burden of hearing impairment. Addressing socio-demographic disparities and improving access to ear and hearing care can substantially mitigate the effects of hearing impairment across Karnali Province.
