# Prevalence and causes of hearing impairment: a cross-sectional study in Karnali Province, Nepal – CORRIGENDUM

**DOI:** 10.1017/S002221512510251X

**Published:** 2025-07

**Authors:** Rishi Bhatta, Sailesh Kumar Mishra, Diego Santana-Hernández, Anil Paudel, Sanju Maharjan, Roshana Kandel, Ranjan Shah, Krishna Khadka, Manisha Budhathoki, Bijaya Guragain, Man Bahadur Kunwar, Manish Gautam

**Affiliations:** 1 ENT, KIST Medical College, Imadol, Nepal; 2 Public health, Anweshan Pvt. Ltd, Lalitpur, Nepal; 3 Nepal Netra Jyoti Sangh, Kathmandu, Nepal; 4 Christoffel-Blindenmission Christian Blind Mission e.V, Germany; 5 CBM Global Nepal, Nepal

**Keywords:** hearing impairment, prevalence, severity of illness, ear diseases, etiology

Upon publication of Bhatta R et al (2025), the ORCID listed for Manish Gautam was incorrect and directed to a different author. The correct ORCID url is https://orcid.org/0009-0001-6059-2197

Additionally the percentage of disabling hearing loss was incorrectly stated within the abstract. The correct sentence should read: *“Among 1946 participants, 38.9 per cent had hearing impairments, including 15.9 per cent with disabling hearing loss, with severity increasing with age.”*

The article has been updated
